# Amyand’s Hernia With Concurrent Appendicitis: A Case Report and Review of the Literature

**DOI:** 10.7759/cureus.22110

**Published:** 2022-02-10

**Authors:** Spencer Probert, Nikhil Nanjappa Ballanamada Appaiah, Akm Shamsul Alam, Narayana Jayachandra Menon

**Affiliations:** 1 General Surgery, Basildon and Thurrock University Hospital, Mid and South Essex NHS Foundation Trust, Basildon, GBR; 2 Colorectal Surgery, Basildon and Thurrock University Hospital, Mid and South Essex NHS Foundation Trust, Basildon, GBR; 3 Cellular Pathology, Basildon and Thurrock University Hospital, Mid and South Essex NHS Foundation Trust, Basildon, GBR

**Keywords:** appendicectomy, laparoscopic appendicectomy, appendicitis, claudius amyand, inguinal hernia, amyand’s hernia

## Abstract

Amyand’s hernia is defined as an inguinal hernia which contains the vermiform appendix. This continues to pose a diagnostic and therapeutic challenge. We herein describe the case of an 11-year-old male with a right-sided Amyand's hernia and concurrent appendicitis. A literature review was also conducted, looking at history, aetiopathogenesis, symptomatology, and management. This case highlights the difficulty clinicians can have in reaching a preoperative diagnosis of Amyand's hernia and selecting the appropriate surgical management.

## Introduction

Amyand’s hernia is a rarely occurring form of inguinal hernia (1% of cases) [1]. Furthermore, concurrent appendicitis within the hernia sac is even rarer (0.1% of cases) [1]. In the picture of Amyand’s hernia, it is also important to realise that the appendix trapped within the hernia can become inflamed, but may not mount an inflammatory response comparable with that of an intra-abdominal acute appendicitis.

Due to the prevalence with which patients are seen with right-sided abdominal pain, and the operator-dependent nature of ultrasonography, it is important that clinicians are aware of this potential diagnosis and have a high index of suspicion. This will allow the appendicitis to be managed before the condition progresses to sepsis or a perforated viscus.

## Case presentation

A normally well, 11-year-old male presented with right-sided abdominal pain and associated vomiting for one day. On initial examination, his abdomen was soft with mild right lower quadrant tenderness, with no other positive findings - including any inguinal swelling or tenderness. The initial accident and emergency (A&E) routine bloods revealed a mildly elevated white cell count (WCC) and a normal C-reactive protein (CRP). Repeat bloods 24 hours later showed the WCC had dropped to within the normal range, and CRP remained normal. It is of note that the patient didn't receive any antibiotic therapy in the interim. The results for these two sets of blood tests can be seen in Table 1.

**Table 1:** Inflammatory markers on initial presentation and 24 hours later. WCC: White cell count; CRP: C-reactive protein.

	Initial Presentation	After 24 hours
WCC (x 10^9^/L)	15.5	9.6
Differential		
Neutrophil Count (x 10^9^/L)	12.74	5.96
Lymphocyte Count (x 10^9^/L)	1.5	2.52
Monocyte Count (x 10^9^/L)	1.24	0.96
Eosinophils Count (x 10^9^/L)	0	0.12
Basophil Count (x 10^9^/L)	0.02	0.03
CRP (mg/L)	<1	3

Due to ongoing tenderness, the patient was sent for an urgent ultrasound scan. This revealed evidence of a lesion with mixed echogenicity within the right iliac fossa. Surrounding this was an indeterminate area of either necrosis or collection, with an area of 4.5 cm x 2.5 cm.

**Figure 1:**
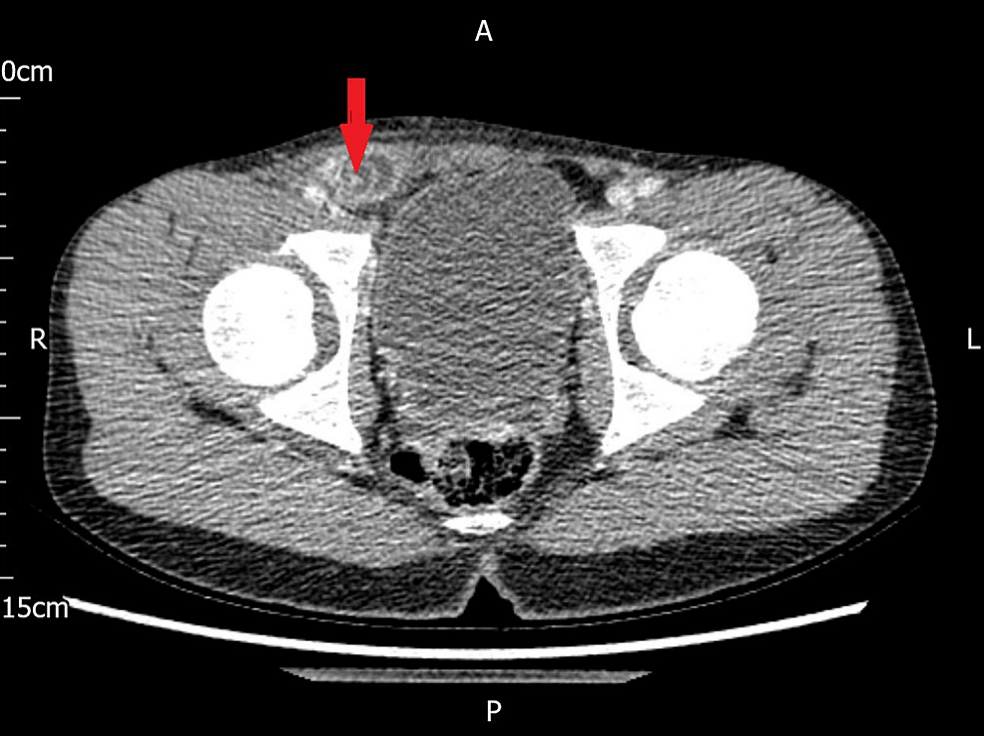
CT axial view showing the right-sided inguinal hernia, containing the appendix.

**Figure 2:**
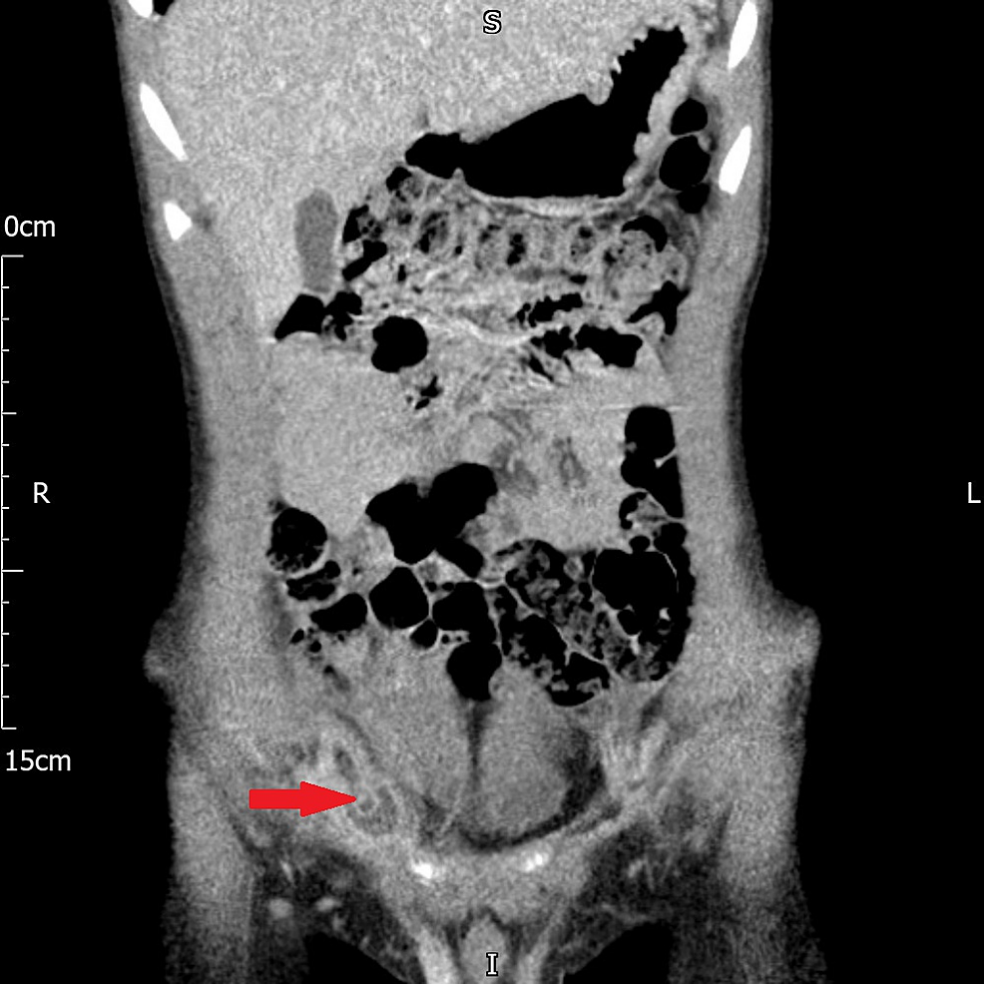
CT coronal view showing the right-sided inguinal hernia, containing the appendix.

On examination, on day 2, the child was comfortable and reported improvement in symptoms. His abdomen was grossly soft and non-tender. There was a focal tender point in the right groin with no cough impulse. Due to ongoing clinical concern and no clear diagnosis, a computerised tomography (CT) scan of the abdomen and pelvis was performed, which confirmed Amyand’s hernia. The report stated - “a small right-sided inguinal hernia containing the tip of the appendix with fluid, and increased peritoneal enhancement suggesting localized infection/inflammation” (Figures 1, 2).

**Figure 3:**
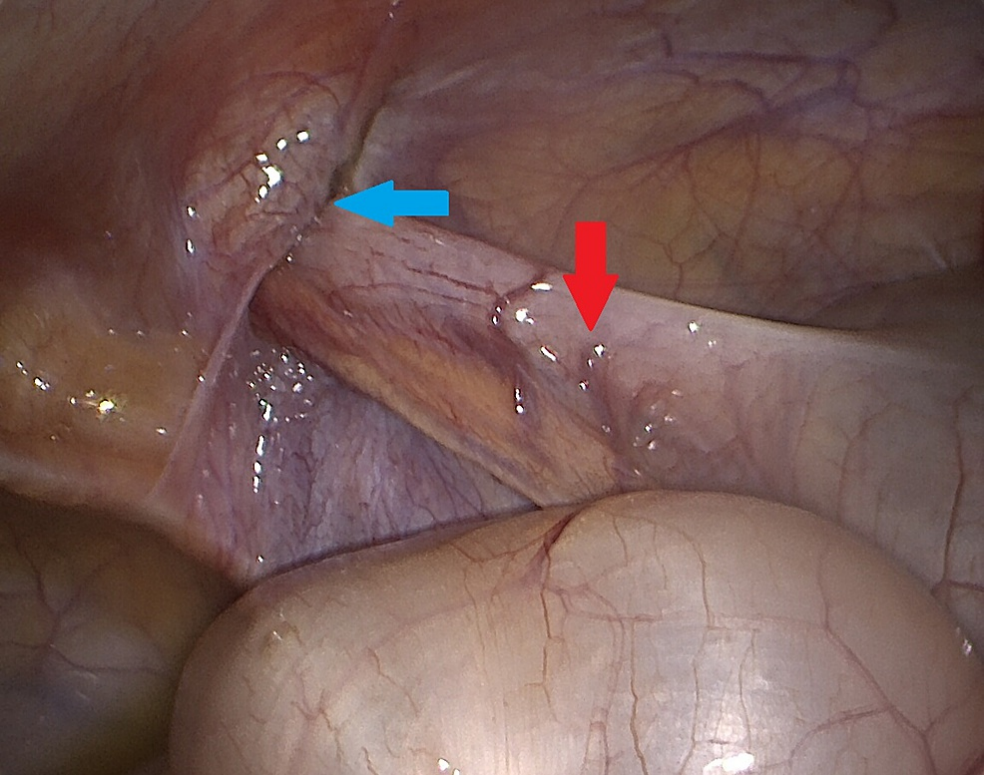
Intraoperative laparoscopic image of the appendix (red arrow) extending into the entrance of the inguinal hernia (blue arrow).

**Figure 4:**
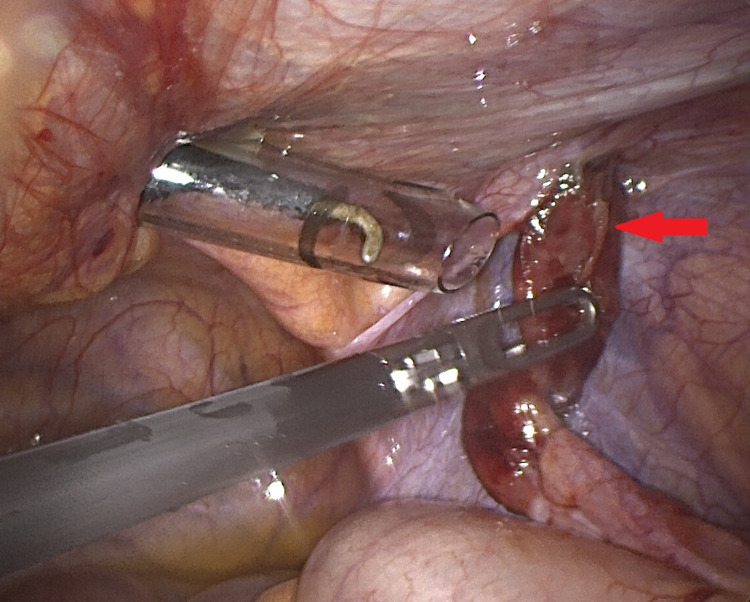
Intraoperative laparoscopic image of the appendix once removed from the hernia. The appendix tip (indicated by arrow) was found to be inflamed.

**Figure 5:**
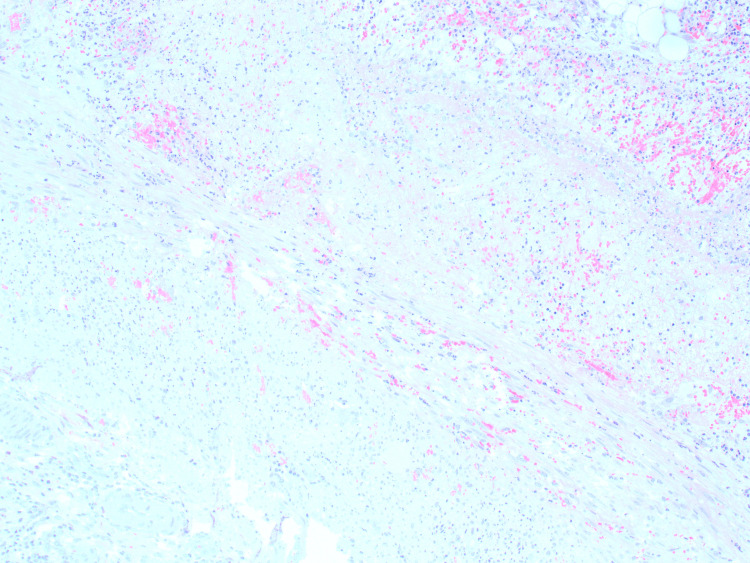
Histology of the tip of the appendix showing significant evidence of acute inflammation.

**Figure 6:**
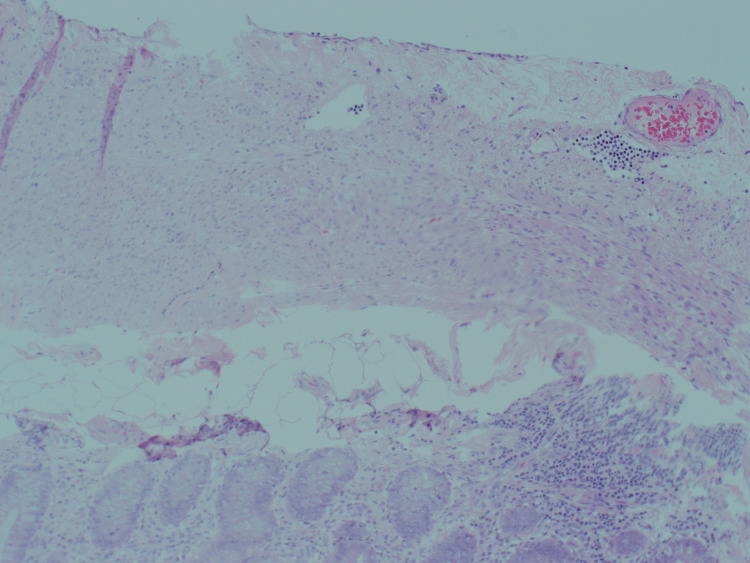
Histology of the base of the appendix showing markedly low evidence of acute inflammation.

The patient underwent a laparoscopic appendicectomy where the appendix was visualized extending into the opening of the right-sided inguinal hernia (Figure 3). On inspection, once removed from the hernia, the appendix was found to be inflamed (Figure 4). The appendix was removed and sent for histology. At the time of operation, it was decided that a concomitant herniotomy would not be performed. The clinical reasoning behind this was that the hernial opening was small and posed a very minimal risk of further viscus incarceration. Histology showed features of acute appendicitis at the tip of the appendix with a normal base (Figures 5, 6). Macroscopically, an inflamed appendix was noted, measuring 40 x 6 mm (at the widest point). Microscopically, there was no evidence of carcinoid tumour, parasites, dysplasia or invasive carcinoma. The patient made an uneventful recovery and was discharged on the first post-operative day.

## Discussion

Amyand’s hernia is a condition where the vermiform appendix is contained within an inguinal hernia sac. The first-ever successful human appendicectomy was performed by Claudius Amyand (1735), wherein the appendix was found to be contained within an inguinal hernia [2]. Subsequently, this combination of inguinal hernia containing the appendix was named after Amyand. This is considered a rare condition, making up only 1% of all inguinal hernias. Furthermore, concurrent appendicitis only occurs in 0.1% of cases [1].

Whilst this condition can present at any age it is more frequently seen in childhood, as a persistent patent processus vaginalis is considered the most common underlying aetiology of an Amyand’s hernia [1]. Despite this, there are reports of Amyand’s hernia in patients as old as 92 [3]. Amyand’s hernia is much more common in male patients. Congenital inguinal hernias have been reported to occur with a 6:1 male to female ratio [4]. Most case reports describe a right-sided Amyand’s hernia, however, there have been some instances where the hernia has been left-sided. In these cases, the hernia is usually associated with congenital intestinal abnormalities (i.e., situs inversus, intestinal malrotation, and mobile cecum) [5]. In a review of 30 reports of Amyand’s hernia, only three were found to be left sided [1].

The mechanism of developing appendicitis with the hernia is not fully understood. There is uncertainty in determining if these are cases of primary appendicitis, coincidentally within the hernia, or if the presence of the appendix within the hernia is responsible for the development of the appendicitis. A number of theories have been presented in the literature. Patoulias et al. conducted a literature review and found four possible theories, these are: incarceration of the appendix inducing inflammation; the development of adhesions between the appendix and the hernial sac causing an irreducible hernia which is susceptible to injury; anterior abdominal muscle contraction (leading to increased intra-abdominal pressure) causing compression and therefore a functional obstruction of the appendix; lastly, primary inflammation of the appendix may lead to an irreducible hernia - this leads to venous stasis and an impaired microcirculation within the appendix, thereby leading to bacterial overgrowth [1, 6].

Clinically these patients usually present as asymptomatic, occasionally with signs of inguinal hernia; inguinal bulge usually reducible. In those that present symptomatically, the patients present with features of an obstructed or strangulated hernia - painful irreducible inguinal bulge, with swelling of overlying tissue and overlying erythema. In the rare case of acute appendicitis (or even perforation of the appendix) within the hernia, the clinical picture is that of perforated intestine within the hernia. There are no specific features to suggest appendicitis and McBurney's sign is typically found to be negative [6, 7]. These factors usually make a preoperative diagnosis of Amyand's hernia very difficult. In some cases, there may be some scrotal swelling and/or pain. Therefore, besides an obstructed or strangulated hernia, other differential diagnoses may include testicular torsion, epididymo-orchitis, hydrocele and inguinal lymphadenitis [7].

Due to the aforementioned preoperative difficulty in diagnosing Amyand’s hernia, most of these patients are diagnosed intraoperatively during the repair of the hernia. Sharma et al. looked at 18 cases of Amyand’s hernia over a 15-year period, of which none of these patients were diagnosed preoperatively. These patients were operated on as emergency cases for either incarcerated or strangulated inguinal hernias and underwent no preoperative imaging [8].

Right-sided abdominal pain is amongst the most common reasons for acute referrals to general surgeons and ultrasonography is usually the first line imaging of choice. Interoperator variability of ultrasonography makes Amyand's hernia challenging to diagnose preoperatively. It is important that clinicians are aware of this condition and the associated clinical ambiguity. Had the authors relied on legacy practice, where acute appendicitis was to be a clinical diagnosis, it is possible that our patient may have gone on to emulate Claudius Amyand’s patient in 1735. When preoperative imaging is performed, the rate of preoperative diagnosis does increase. Okur et al. looked at 21 cases of Amyand’s hernia at a single center. Ultrasound was done in 12 of the cases (57.1%) and diagnosed 75% (9/12) with Amyand’s hernia preoperatively [9].

Ultrasound findings usually include a non-compressible structure within the inguinal hernia sac (when there is concurrent appendicitis, this structure typically has wall thickening and hyperaemia). CT imaging of Amyand’s hernia usually reveals a blind-ending structure within the hernial sac, originating at the caecum. Similarly, to the ultrasound findings, when there is concurrent appendicitis there will be some degree of wall thickening, hyperaemia and fat stranding [1]. It is pertinent that surgeons and radiologists collaborate on complex decisions with regards to cross-sectional imaging in children. When the ultrasound findings are not conclusive, it would be prudent to use cross-sectional imaging in these situations. Pre-operative diagnosis of Amyand’s hernia is essential in planning surgical management and approach, especially in children - where the use of laparoscopic surgery is only now gaining wide-spread acceptance.

Since most cases of Amyand’s hernia are diagnosed intraoperatively, it is hard to standardize an approach to treatment and usually left to the discretion of the operating surgeon. Losanoff and Basson, in 2008, proposed a classification system for the management of Amyand’s hernia [10]. This is summarised in Table 2. An additional category, known as the “Rikki Modification” has since been added. This additional category deals with cases where there is an incisional hernia, in which the appendix lies. Within this category a normal appendix is managed as type 1; an acute appendicitis is managed by appendicectomy through the hernia and then subsequent hernia repair with a primary closure; and finally a peritonitic abdomen with sepsis is managed as type 4 [1].

**Table 2:** Losanoff and Basson classification with proposed management of Amyand’s hernia.

Type	Amyand’s hernia with...	Proposed Management
1	Normal appendix.	Hernia repair, with mesh.
2	Acute appendicitis with no abdominal sepsis.	Laparoscopic appendicectomy with primary hernia repair.
3	Acute appendicitis with abdominal sepsis.	Open appendicectomy with primary hernia repair.
4	Acute appendicitis with concurrent intra-abdominal pathology.	Open appendicectomy with primary hernia repair, with investigation and management of respective intra-abdominal pathology as relevant.

## Conclusions

This case highlights the diagnostic challenge posed by Amyand's hernia. With the assistance of imaging, a preoperative diagnosis was reached which enabled an appropriate and planned surgical approach. Clinicians should have a high index of suspicion for Amyand’s hernia and a low threshold for investigating further with imaging. Due to the appendix being isolated from the abdomen, blood tests may return as normal and can therefore be very misleading in a suspected appendicitis. With the use of imaging (ultrasound & low dose CT), a pre-operative diagnosis could be made for a significant number of patients. This would allow for a better visualization of the appendix and inguinal canal, and possibly a better surgical outcome.

## References

[1] Patoulias D, Kalogirou M, Patoulias I (2017). Amyand's hernia: an up-to-date review of the literature. Acta Medica (Hradec Kralove).

[2] Amyand C (1735). VIII. Of an inguinal rupture, with a pin in the appendix coeci, incrusted with stone; and some observations on wounds in the guts. Philos Trans R Soc Lond.

[3] Anagnostopoulou S, Dimitroulis D, Troupis TG (2006). Amyand's hernia: a case report. World J Gastroenterol.

[4] Brainwood M, Beirne G, Fenech M (2020). Persistence of the processus vaginalis and its related disorders. Australas J Ultrasound Med.

[5] Diego Alonso E, Ayuso Velasco R, Cebrián Muiños C, Moreno Zegarra C, Liras Muñoz J (2020). Left Amyand's hernia associated with omphalocele (Article in English, Spanish). Cir Pediatr.

[6] Singhal S, Singhal A, Negi SS (2015). Amyand's hernia: rare presentation of a common ailment. Case Rep Gastrointest Med.

[7] Fernando J, Leelaratna S (2002). Amyand’s hernia. Ceylon Med J.

[8] Sharma H, Gupta A, Shekhawat NS, Memon B, Memon MA (2007). Amyand's hernia: a report of 18 consecutive patients over a 15-year period. Hernia.

[9] Okur M, Karaçay Ş, Uygun İ, Topçu K, Öztürk H (2013). Amyand’s hernias in childhood (a report on 21 patients): a single-centre experience. Pediatr Surg Int.

[10] Losanoff JE, Basson MD (2008). Amyand hernia: a classification to improve management. Hernia.

